# Treatment of severe tricuspid regurgitation induced by permanent pacemaker lead: Transcatheter tricuspid valve replacement with the guidance of 3-dimensional printing

**DOI:** 10.3389/fcvm.2023.1030997

**Published:** 2023-03-22

**Authors:** Yu Mao, Yang Liu, Xin Meng, Yanyan Ma, Lanlan Li, Mengen Zhai, Ping Jin, Fanglin Lu, Jian Yang

**Affiliations:** ^1^ Department of Cardiovascular Surgery, Xijing Hospital, Air Force Medical University, Xi’an, China; ^2^ Department of Ultrasound Medicine, Xijing Hospital, Air Force Medical University, Xi'an, China; ^3^ Department of Cardiovascular Surgery, Changhai Hospital, Naval Military Medical University, Shanghai, China

**Keywords:** tricuspid regurgitation, transcatheter tricuspid valve replacement, 3-Dimensional printing, LuX-Valve, permanent pacemaker implantation

## Abstract

**Background:**

Lead-induced tricuspid regurgitation is one of the complications after permanent pacemaker implantation (PPI) and refers to tricuspid regurgitation (TR) caused by the lead in the right ventricle (RV).

**Objectives:**

To study the clinical characteristics of severe TR after PPI and the effect of transcatheter tricuspid valve replacement (TTVR) with the guidance of 3-dimensional (3D) printing.

**Methods:**

This study was a single-center, descriptive study. Six patients with severe TR after PPI were enrolled in Xijing Hospital from January 2020 to May 2020. Before TTVR, the 3D printed tricuspid valve (TV) model was used for evaluation in the bench test. LuX-Valve was implanted under the guidance of TEE and x-ray fluoroscopy, and all patients underwent transatrial access. Six patients’ data were collected at baseline, before discharge, and 6 months, 1 year and 2 years after TTVR.

**Results:**

The LuX-Valve was successfully implanted in 6 patients, TR was significantly reduced to ≤2+, and no deaths or cardiopulmonary bypass occurred during procedures. Three cases were caused by TV expansion: Patient #4 had TR caused by lead adhesion to TV, Patient #2 had TR caused by lead winding, and Patient #6 had TR caused by lead impingement on TV. During the 2-year follow-up, TTE revealed that 5 patients had no/trace regurgitation, and one patient (Patient #5) had mild regurgitation. All 6 patients (100.0%) reached primary endpoints.

**Conclusion:**

TTVR guided by 3D printing is safe and effective in the treatment of severe TR associated with permanent pacemaker lead, providing prospects and possibilities for the precise treatment of TV-related diseases.

**Clinical Trial Registration**: ClinicalTrials.gov Protocol Registration System (NCT02917980).

## Introduction

The transvenous system of lead implantation in the right heart is the cornerstone of permanent pacemaker implantation (PPI). Due to the increasing number of PPIs and the enhanced understanding of tricuspid valve (TV) diseases associated with pacemaker lead, there has been an increasing amount of research on this topic (1, 2). Studies have found that lead-induced tricuspid regurgitation (LITR) is not uncommon, with an incidence of 7.3%–39.0%. In particular, LITR has a significant impact on cardiac function and poor long-term prognosis (2). LITR is one of the complications after PPI and refers to tricuspid regurgitation (TR) caused by the lead in the right ventricle (RV). Some studies have shown that LITR accounts for 25% of TR (3, 4), which may increase the long-term mortality of patients (5). The pathogenesis of LITR can be divided into mechanical and electrophysiological mechanisms. Among mechanical mechanisms, the most important factor is lead impingement with the leaflets of the TV, in which the posterior valve and septal valve are most easily affected (6). However, electrophysiological mechanisms are mainly associated with the dyssynchronization of atrioventricular contraction (7). Therefore, for patients with PPI, LITR should be suspected once there have been several symptoms that indicate right heart failure. LITR of moderate to severe degree has a longer process to aggravate, and factors such as volume load, leaflet traction, and poor leaflet al.ignment may aggravate TR (8). In the treatment of severe LITR, diuretics are the basic means (9). However, when LITR progresses to right cardiac dysfunction, there is a high risk of lead removal, which usually requires surgical treatment (2). Previously, studies have reported that surgery on the TV among individuals with severe LITR has not led to irreversible right heart failure and not only reduces the risk of procedures but also prevents the continuous deterioration of right cardiac function caused by severe LITR and improves the survival rates of patients (4). In recent decades, transcatheter tricuspid valve replacement (TTVR) has become one of the research hotspots in cardiovascular medicine. Several interventional devices for different anatomical structures of the TV have been used clinically. Early reports from these devices have shown varying degrees of reduction in TR (7, 10–13). LuX-valve (Ningbo Jenscare Biotechnology, Ningbo, China) is a TTVR device unrelated to radial force that has been reported to be successfully implanted in severe TR patients (14). However, TTVR after PPI has rarely been reported. This study reported the single-center experience of TTVR to treat patients with severe TR after PPI and explored the guiding role of 3D printing in the preprocedural plan of TTVR.

## Methods

### Study population

This study was a single-center, observational study. From September 2020 to May 2021, 6 patients with severe TR after PPI [4 women; 62.0 (58.0, 71.0) years] were enrolled in this study. The inclusion criteria were as follows: prior PPI and the presence of severe TR confirmed by transthoracic echocardiography (TTE) (15). The exclusion criteria were as follows: other secondary causes of TR were excluded, including infective endocarditis, prior operations in the left heart valve, congenital heart disease, TR prior to PPI, and rheumatic TV diseases. This study was conducted in accordance with the principles of the Declaration of Helsinki and related ethical requirements. Ethical registration was registered in ClinicalTrials.gov Protocol Registration System (NCT02917980).

### Preprocedural imaging

**Figure 1:**
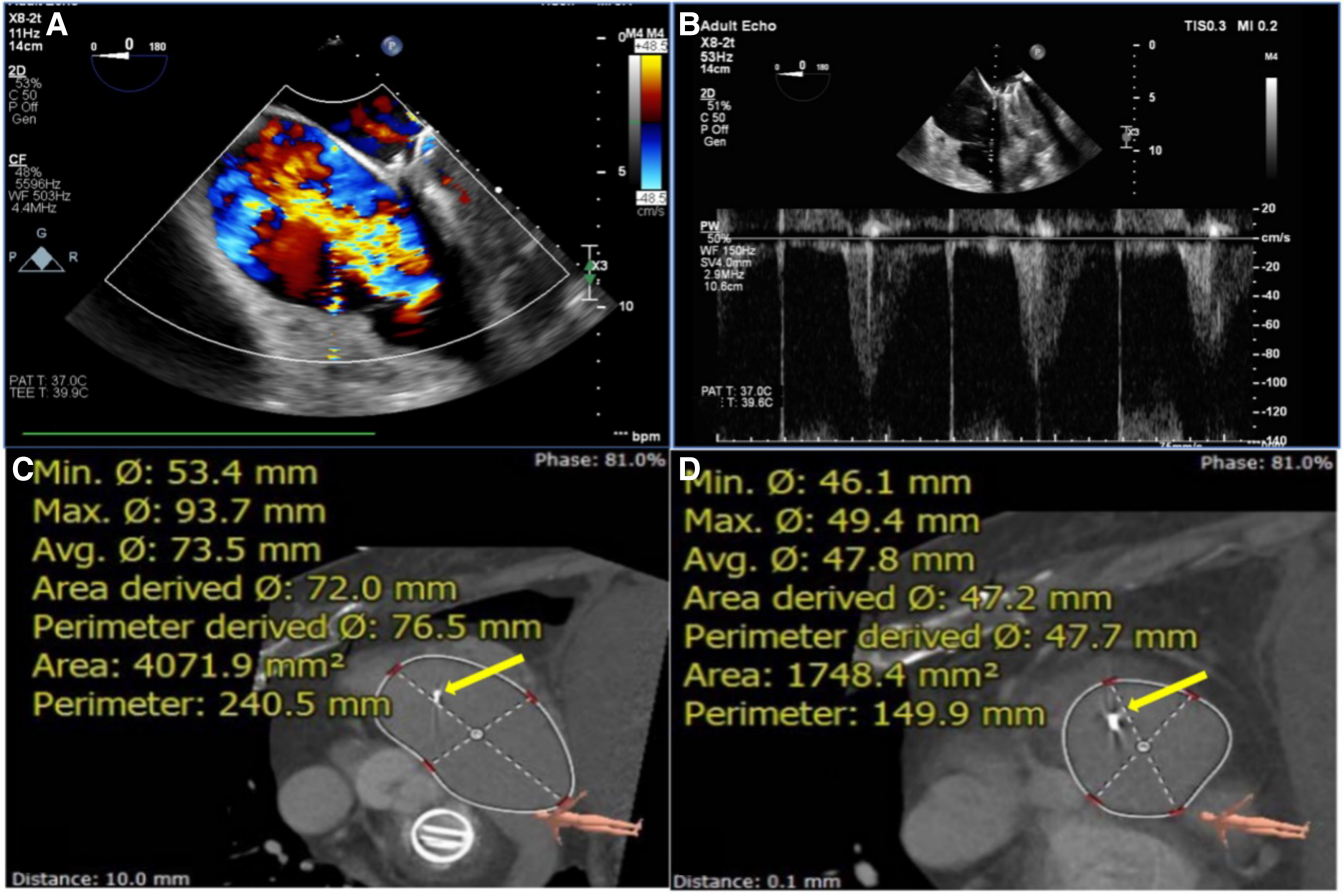
Assessment of TEE and CTA before TTVR evaluated the status of TV (e.g., data for patient #6) assessment of RA and RV using circle cardiovascular imaging CVI42 software. (**A**) TEE showed large regurgitation on TV. (**B**) The maximum velocity of TV was up to 1.1 m/s. (**C, D**) The area of the RA was 4071.9 mm^2^, and the area of the RV was 1748.4 mm^2^. The yellow arrows indicate the lead of the permanent pacemaker. TEE, transesophageal echocardiography; CTA, computed tomography angiography; TTVR, transcatheter tricuspid valve replacement; TV, tricuspid valve; RA, right atrium; RV, right ventricle; TA, tricuspid annulus.

Coronary angiography was used to exclude severe coronary artery diseases, invasive RV catheterization was used to evaluate the hemodynamics of the right heart, and gated cardiac computed tomography (CCT) was used to evaluate anatomical structures. Functional TR is considered to be a disease that depends not only on the sizing and shape of TV but also on the function of the RV, ventricular septal displacement, and pulmonary artery pressure (PAP) (16). Transthoracic echocardiography (TTE) and transesophageal echocardiography (TEE) were both performed in all patients before the procedures to assess RV and TV functions (Figures 1A–D).

### Device description

**Figure 2:**
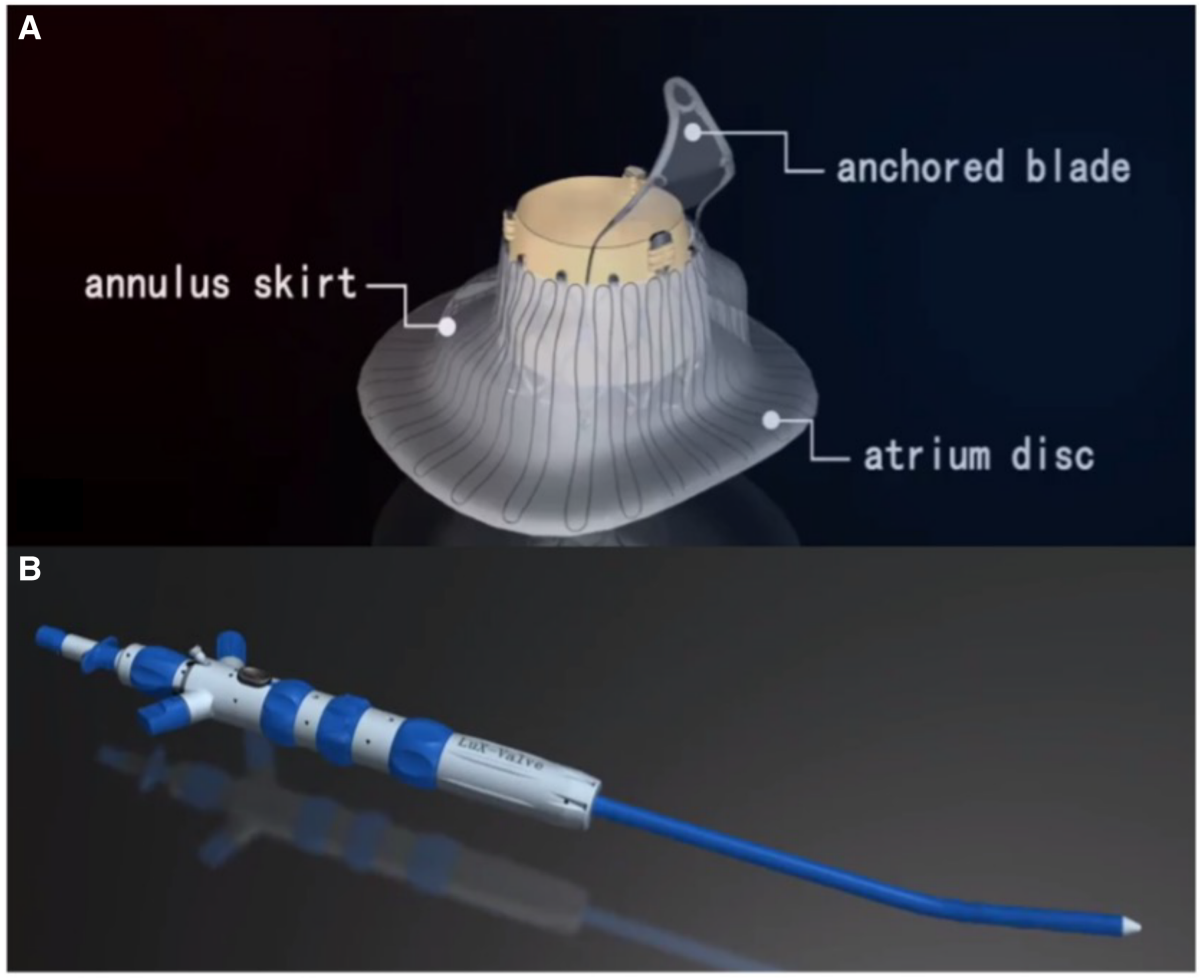
Introduction of LuX-valve. (**A, B**) LuX-valve (Ningbo Jenscare Biotechnology, Ningbo, China) consists of a biological valve stent, three valve lobules, and a steerable delivery system. The bioprosthesis is implanted *via* the RA approach and fixed in the TA with its own unique anchoring device, independent of the radial support force. It consists of an atrium disc, annulus skirt and anchored blade.

LuX-Valve consists of a biological valve stent, three valve lobules, and a steerable delivery system (Figure 2). It is funnel-shaped and consists of four parts: (1) bovine pericardial valve leaflets of GeniGal® anti-calcification treatment to improve durability; (2) pioneered interventricular anchor (IVA) to solve the problem of TV fixation; (3) adaptive positioning of leak-proof ring, with no pressure on the tissues around TV; (4) the clamping device of the anterior valve, with both positioning and ventricular septal anchoring function to form stable anchoring; (5) six types of device sizing, three types of plate skirt to suit all kinds of the pathological anatomical structures of TV.

### Reconstruction of 3d printed models

**Figure 3:**
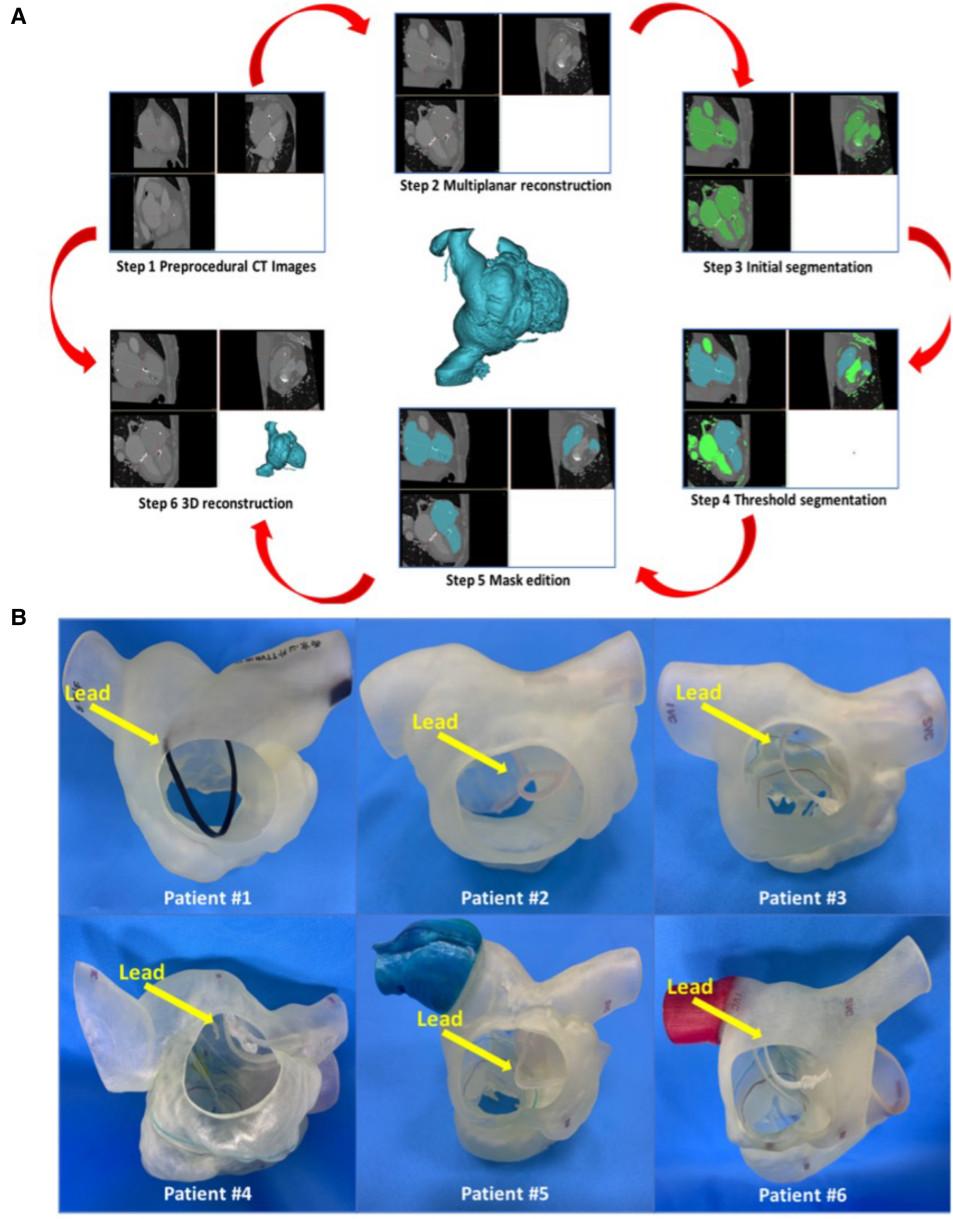
Preoperative 3D reconstruction and 3D-printed models of all 6 patients to help surgeons understand anatomical structures and make an accurate surgical plan. (**A**) The process of 3D reconstruction using Materialise Mimics version 21.0 (Materialise, Leuven, Belgium). (**B**) 3D-printed models of all 6 patients’ right cardiac systems in the RA view. The yellow arrow heads to the lead of the permanent pacemaker. RA, right atrium; 3D, 3-dimensional.

**Figure 4:**
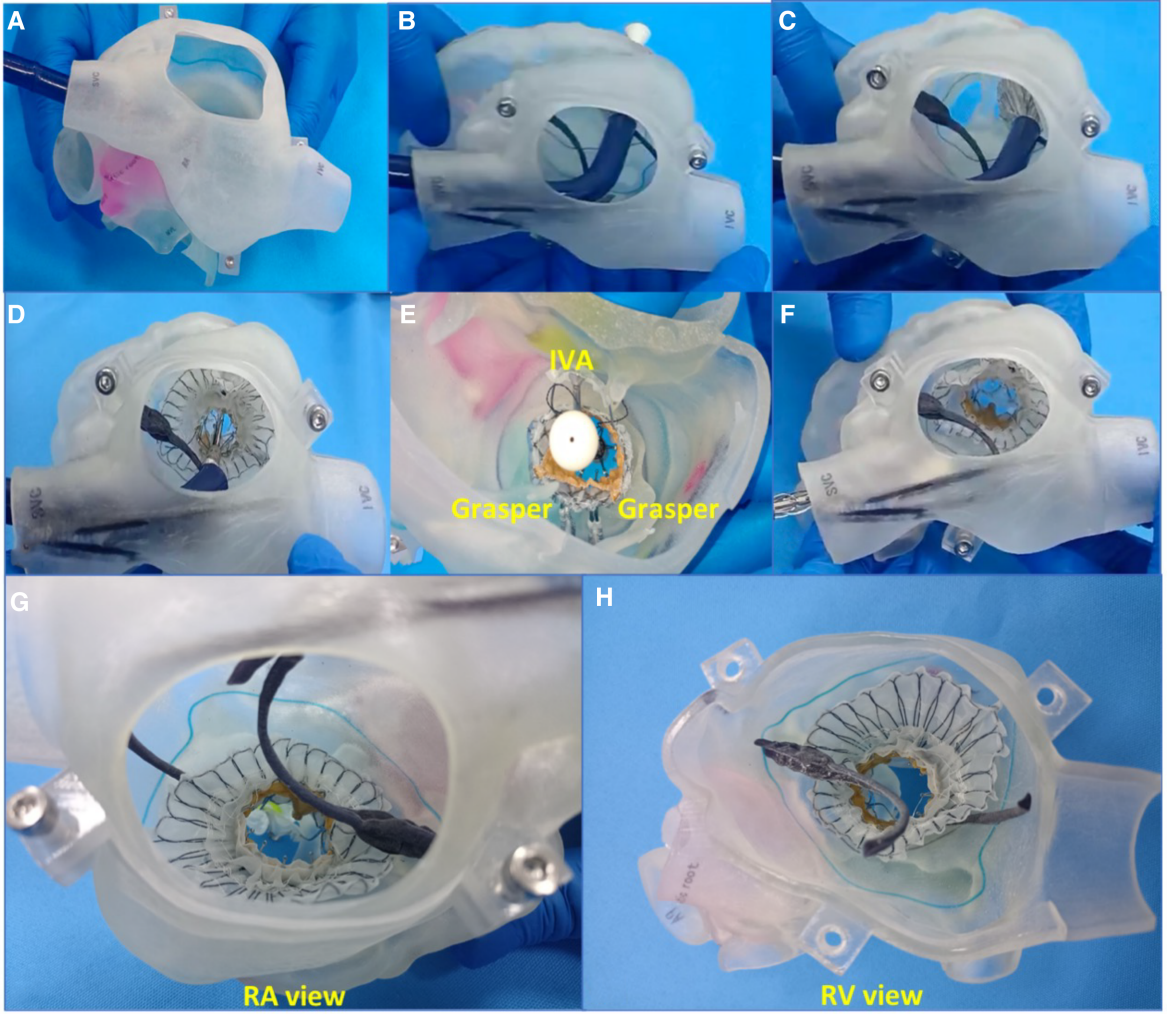
Simulation of TTVR using a 3D-printed model on the bench test (e.g., data for patient #6). (**A**) The delivery system was entered into the RA through the superior vena cava. (**B**) The delivery system was positioned to the TA, and the depth and coaxiality were adjusted. (**C**) The atrium disc began to be released. (**D**) The atrium disc was fully released. (**E**) IVA and two clamping keys were released in turn. (**F**) The delivery system was withdrawn. (**G**) RA view of TV after simulation. (**H**) RV view of TV after simulation. TTVR, transcatheter tricuspid valve replacement; RA, right atrium; TA, tricuspid annulus; RV, right ventricle; IVA, interventricular anchor; TV, tricuspid valve.

First, the patients' CT data were imported into Materialise Mimics version 21.0 (Materialise, Leuven, Belgium), and three orthogonal slices (coronal, sagittal and cross section) were created using interactive multiplane imaging reconstruction. After the comparison and confirmation, the contour area was reconstructed to obtain the initial 3D model of RV, and the collected images were converted to the standard format of Digital Imaging and Communication of Medicine (DICOM) for storage (Figure 3A). Second, a comprehensive reconstruction of RV morphology was performed using Materialise 3-matic software (Materialise, Leuven, Belgium). Different parts of the digital model were distinguished by different colors to represent the multidimensional structure information of each part. Finally, the digital model is exported to Standard Tessellation Language (STL) format. The STL files were imported into the Polyjet 850 multimaterial full-color 3D printer (Stratasys, Inc., Eden Prairie, MN, USA) for printing, selecting different materials to match different tissues (Figure 3B). The main steps of TTVR were simulated in the cath laboratory for all 6 patients (Figure 4).

### Procedure steps

**Figure 5:**
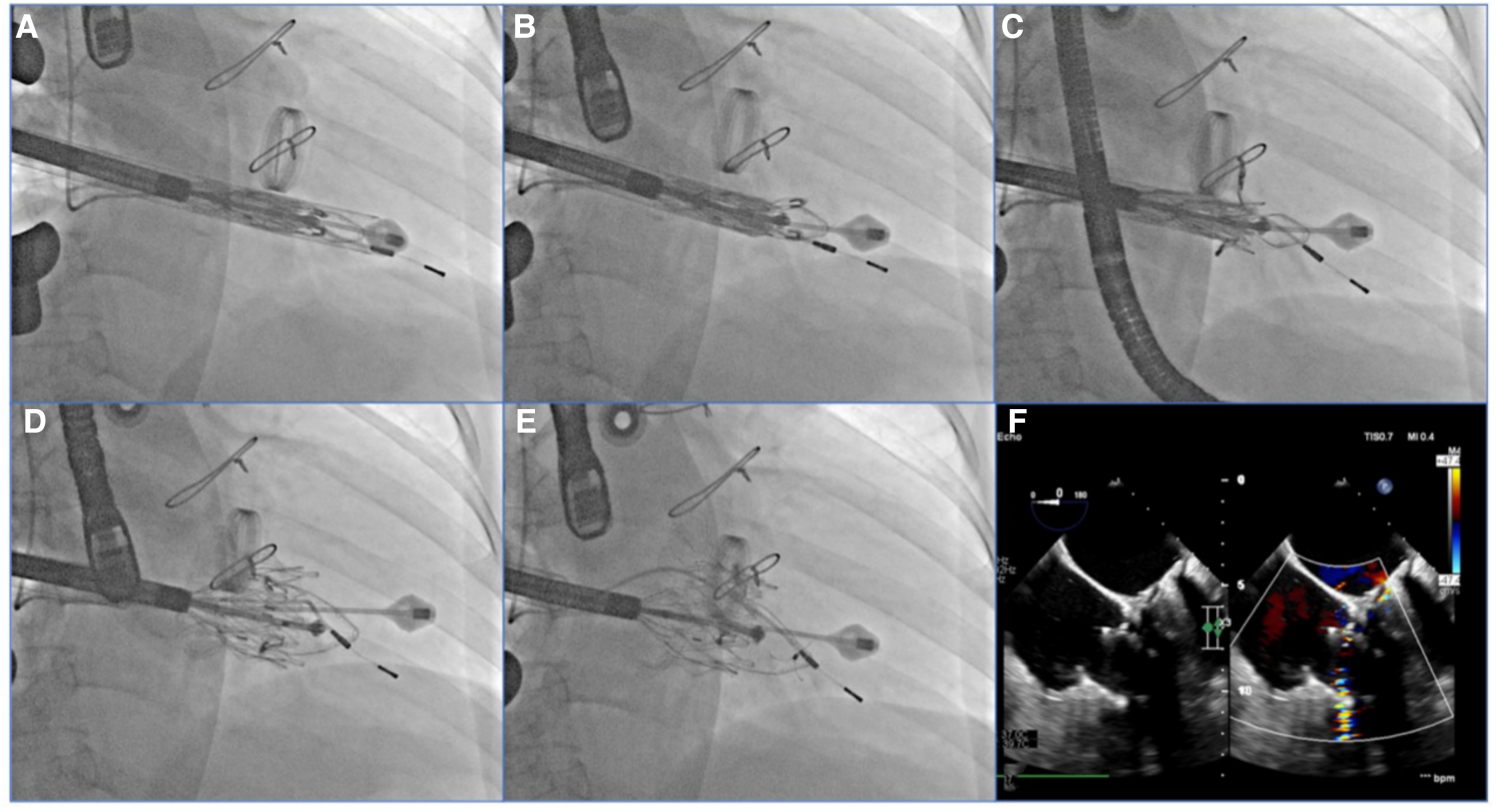
TEE and DSA were guided for TTVR. (**A**) DSA showed severe TR on TV. (**B**) The LuX-Valve system guided by TEE was used to deliver the prosthesis to the tricuspid annulus through the right intercostal approach. (**C**) The delivery system first released IVA and two graspers, and TEE guided the anterior leaflet of the tricuspid valve to be clamped by the grasper. (**D**) The annulus skirt and atrium disc were released in turn, and the position of the implant was adjusted by TEE guidance to ensure that there was no obvious paravalvular leakage. (**E**) The prosthesis was completely released after fixation with IVA. (**F**) Postoperative TEE showed that TR disappeared immediately. TEE, transesophageal echocardiography; DSA, digital subtracted angiography; TR, tricuspid regurgitation; TV, tricuspid valve; IVA, interventricular anchor.

After general anesthesia, TV was entered with a right minimally invasive thoracotomy and through the right atrial (RA) path. TEE and x-ray fluoroscopy were used for the guidance. TEE was mainly used to guide catheter delivery, valve release, and adjustment of intraoperative valve position. A coronary artery guide wire was placed in the right coronary artery to help determine the annulus plane of the TV. Systemic heparinization was performed to achieve an activated coagulation time of >200 s, and then, 4–0 prolene sutures with felt sheets were used with a double purse suture in the RA. The delivery catheter was placed into the RV under the guidance of TEE and x-ray fluoroscopy. With the adjustment, the angle of the catheter was adjusted to ensure that the catheter was coaxial and centered with the ring. When the catheter was under the loop of approximately 5 cm, the IVA and two clamping keys of the anterior lobes were released in turn by adjusting the knob system on the catheter. Then, the clamping keys were positioned properly under the anterior lobe, and the entire delivery system was gently retracted so that the clamping keys hooked the anterior lobe. The atrial plate was released, IVA was deployed, and the anchor pin was inserted into the septum for fixation. Finally, the catheter was withdrawn and removed, heparin was neutralized, and the atrial incision was closed (Figure 5).

### Data collection

Baseline data were collected by consulting the electronic medical record system. The operative time, valve implantation time, x-ray fluoroscopy time and RA pressure were recorded. The valve implantation time was defined as the time from catheter entry into the RA to withdrawal from the RA. In addition, data during hospitalization were collected (including ICU stay time, in-hospital days, and postoperative TTE data).

### Follow-up

Follow-up data were collected from enrolled patients at baseline, before discharge, and 6 months, 1 year and 2 years after surgery. Primary endpoints included successful surgery. Successful surgery was defined as successful implantation of the valve and removal of the delivery system and the correct and stable placement of the prosthesis, with no serious or life-threatening adverse events during surgery. The function of TV is recovered satisfactorily [TR severity is reduced by ≥2, TV pressure gradient (PG) ≤ 6 mmHg], and there was no cardiovascular mortality, implant displacement, valve failure, or other major adverse events related to the device (including myocardial infarction, embolism, conduction disturbances, and new trans-ventricular septal shunt).

### Statistical analysis

Continuous variables were reported as the median (25th and 75th percentile), while classified variables were expressed by frequency and percentage. A two-tailed *P* value of <0.05 was considered statistically significant. All statistical analyses were conducted by using Statistical Package for Social Sciences (SPSS, Chicago, Illinois, USA) version 26.0.

## Results

### Baseline data

The baseline clinical characteristics of the 6 patients are listed in Table 1. The preoperative time of PPI was 23 (7, 82) months, and the indications of PPI included left bundle branch block (LBBB) in 2 patients (33.3%), right bundle branch block (RBBB) in 3 patients (50.0%), and sick sinus syndrome (SSS) in 3 patients (50.0%). The status and management of leads in patients are listed in Table 2. All 6 patients (100.0%) were implanted with an atrioventricular pacemaker. Although diuretic therapies were administered, all patients had typical symptoms of severe right heart failure (100.0%). The causes of TR were tricuspid annular (TA) expansion (50.0%), lead adhesion (16.7%), lead impingement (16.7%) and lead winding (16.7%). Baseline echocardiographic parameters are listed in Table 3. All 6 (100.0%) patients had severe TR at baseline. Preoperative right heart catheterization showed that the mean pulmonary artery pressure (mPAP) of the included patients was 25.5 (18.0, 32.0) mmHg, and 3 patients (50.0%) had pulmonary hypertension (PH) before surgery. In addition, the New York Heart Association (NYHA) functional class in all 6 patients (100.0%) was III/IV, with a European system for cardiac operative risk evaluation (EuroSCORE II) of 9.9 (7.9, 11.6) % and Society of Thoracic Surgeons (STS) score of 10.1 (8.1, 12.4) %, indicating a high risk of cardiopulmonary bypass.

**Table 1:** Baseline patient characteristics.

Characteristics	Patient #1	Patient #2	Patient #3	Patient #4	Patient #5	Patient #6
Age (years)	60	71	62	66	62	58
Sex	Male	Female	Female	Female	Male	Female
Body mass index (kg/m^2^)	24.3	21.8	19.5	20.4	25.7	21.0
NYHA class	IV	IV	III	III	IV	IV
STS score (%)	11.3	8.1	9.3	8.7	12.4	10.9
EuroSCORE II (%)	10.7	7.9	9.2	8.4	11.6	10.5
6MWT (m)	190.0	220.0	205.0	215.0	165.0	190.0
KCCQ	29.0	34.0	32.0	32.0	27.0	29.0
Clinical symptoms	Peripheral edema, Ascites	Peripheral edema	Peripheral edema	Peripheral edema	Peripheral edema, Ascites	Peripheral edema, Ascites
**Blood sampling**						
Hemoglobin (g/L)	101.8	106.8	98.5	103.6	96.3	91.4
Albumin (g/dl)	3.6	4.0	3.4	3.6	3.2	3.2
Bilirubin (mg/dl)	0.9	1.3	1.1	1.2	0.9	0.8
Creatinine (mg/dl)	1.2	0.8	0.9	0.9	1.2	1.1
eGFR (ml/min)	50.6	63.2	55.7	58.3	44.9	49.1
BNP (pg/ml)	274.8	143.0	202.1	161.6	331.5	308.7
NT-proBNP (pg/ml)	910.2	589.7	775.0	647.6	1009.1	963.4
Alanine transaminase (U/L)	24.3	11.7	17.5	13.1	21.2	22.6
Aspartate transaminase (U/L)	41.0	18.5	20.9	25.2	37.7	35.8
INR	0.9	1.9	1.5	1.7	0.9	1.2
**Right heart catheterization**						
sPAP (mm Hg)	41.0	35.0	39.0	33.0	48.0	41.0
mPAP (mm Hg)	29.0	19.0	24.0	18.0	32.0	27.0
Pulmonary hypertension^*^	Yes	No	No	No	Yes	Yes
**Comorbidities**						
Diabetes	Yes	No	No	Yes	Yes	No
Atrial fibrillation	No	No	No	No	No	No
SSS	Yes	No	No	No	Yes	Yes
LBBB	No	Yes	No	No	No	Yes
RBBB	Yes	No	Yes	No	Yes	No
Coronary artery disease	No	No	No	No	Yes	Yes
Chronic obstructive pulmonary disease	Yes	No	No	No	No	No
Chronic kidney disease^†^	Yes	Yes	Yes	Yes	Yes	Yes
Severe liver disease^‡^	Yes	No	No	No	Yes	Yes
Prior gastrointestinal hemorrhage	No	No	No	No	No	No
Prior stroke/TIA	Yes	No	No	No	No	No
Malignancy	No	Yes	No	No	No	No
**Previous cardiac intervention**						
Coronary artery bypass grafting	No	No	No	No	Yes	Yes
Left-sided valvular surgery	No	Yes	Yes	Yes	Yes	No

Values are presented as *n* (%) or median (25th, 75th percentile).

BNP, B-type natriuretic peptide; eGFR, estimated glomerular filtration rate; ICD, implantable cardioverter defibrillator; KCCQ, Kansas City cardiomyopathy questionnaire; STS, society of thoracic surgeons; EuroSCORE, European system for cardiac operative risk evaluation; INR, international normalized ratio; mPAP, mean pulmonary artery pressure; 6MWT, 6 min walk test; NT-proBNP, N-terminal pro-B-type natriuretic peptide; NYHA, New York heart association; PPM, permanent pacemaker; TIA, transient ischemic attack; sPAP, systolic pulmonary artery pressure; RBBB, right bundle branch block; LBBB, left bundle branch block; SSS, sick sinus syndrome.

^*^ mPAP ≥25 mm Hg.

^†^ Defined as eGFR <60 ml/min.

^‡^ Defined as MELD-albumin score >12.

**Table 2:** Status and management of leads in patients with a transvenous pacing system who underwent TTVR.

Status of patients	Patient #1	Patient #2	Patient #3	Patient #4	Patient #5	Patient #6
History of PPI	1 time	1 time	1 time	1 time	2 times	1 time
Preoperative time of PPI (months)	12	82	16	47	7	30
Type of permanent pacemaker	Dual-chamber pacemaker	Dual-chamber pacemaker	Dual-chamber pacemaker	Dual-chamber pacemaker	Dual-chamber pacemaker	Dual-chamber pacemaker
Number of RV leads	1	1	1	1	1	1
Causes of TR	TA expansion	Leads winding	TA expansion	Leads adhesion	TA expansion	Leads impingement

PPI, permanent pacemaker implantation; RV, right ventricular; TR, tricuspid regurgitation; TV, tricuspid valve; TA, tricuspid annular.

**Table 3:** Baseline echocardiographic and computed tomography parameters.

Echocardiographic parameters	Patient #1	Patient #2	Patient #3	Patient #4	Patient #5	Patient #6
RV basal diameter (mm)	66.5	55.3	52.8	49.3	62.0	57.8
RV mid diameter (mm)	48.8	43.0	41.5	38.0	50.5	46.3
Fractional area change (%)	41.1	38.0	37.5	34.6	39.7	42.7
TAPSE (mm)	15.5	13.3	12.7	11.6	14.4	13.5
RV systolic TDI (cm/s)	11	8	10	8	12	12
RA volume index (mL/m^2^)	104.3	80.6	92.0	84.2	115.9	112.1
EROA PISA (mm^2^)	72.0	68.0	71.0	67.0	75.0	70.0
LVEF (%)	54.0	46.0	44.0	46.0	43.0	52.0
Transient regurgitation volume (ml)	83.5	58.6	77.3	72.6	100.8	80.7
TR severity^*^	Severe	Severe	Severe	Severe	Severe	Severe
TR velocity (m/s)	3.46	1.89	2.86	2.10	3.55	3.29
**Computed tomography parameters**						
TA maximum diameters (mm)	54.3	49.9	47.4	45.6	52.6	50.3
TA minimum diameters (mm)	42.8	40.2	39.5	38.1	42.0	41.1

EROA, effective regurgitation orifice area; LVEF, left ventricular ejection fraction; LVIDD, left ventricular internal dimension in diastole; LVIDS, left ventricular internal dimension in systole; PISA, proximal isovelocity surface area; RA, right atrium; RV, right ventricular; TAPSE, tricuspid annular plane systolic excursion; TDI, tissue Doppler imaging; TR, tricuspid regurgitation.

^*^ TR severity is classified into mild, moderate, severe, very severe and extremely severe (21).

### Intraoperative and hospitalization data

**Figure 6:**
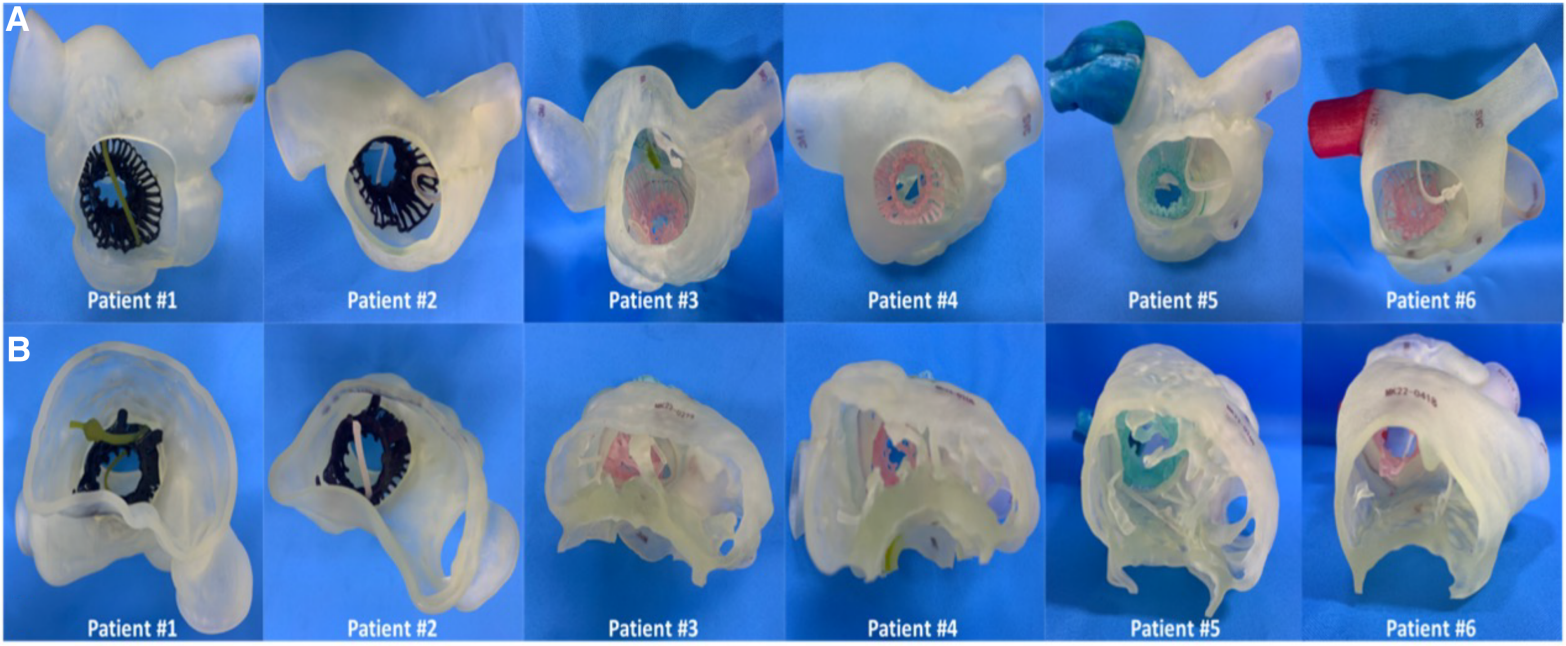
The postoperative 3D reconstruction and 3D-printed models of all 6 patients verified the position of the stented valve, and the lead remained attached to the RV with no change in threshold after the valve was implanted. (**A**) 3D printed model of the patient's right cardiac system in the RA view. (**B**) 3D printed model of the patient's right cardiac system in the RV view. RA, right atrium; RV, right ventricle; 3D, 3-dimensional.

The intraoperative and hospitalization details are shown in Table 4. All patients were treated 3 to 5 days before surgery and, if tolerated, were treated with intravenous diuretics to reduce weight and improve peripheral edema. Surgical success was achieved in all patients (100%), with the individual valves in place in all cases. The median procedural time was 135 (120, 150) min, the device time was 8.5 (7.0, 12.0) min, and no persistent ventricular arrhythmias, atrioventricular block or cardiac rupture occurred. After the procedures, TEE detected mild paravalvular leakage in Patient #5 (16.7%), possibly due to leaflet damage during valve crimping. The remaining 5 patients (83.3%) had no/trace regurgitation. The postoperative ICU days were 2.3 (1.0, 6.0) days, and the postoperative hospitalization days were 10.5 (8.0, 17.0) days. In patients with no preexisting renal impairment, RV angiography was performed to confirm the position and function of the implanted valve. Before discharge, CT confirmed the position and fixation details of the prosthesis. computed tomography angiography (CTA) data of all 6 patients were used for 3D reconstruction, and 3D models were printed to verify postoperative morphology and function (Figure 6). In addition, there were no pulmonary embolisms, cerebrovascular events or new conduction blocks during hospitalization. All discharged patients were treated with anticoagulants. All patients had a ≥2 grade reduction in TR severity from preoperative levels.

**Table 4:** Intraoperative and in-hospital outcomes.

Intraoperative outcomes	Patient #1	Patient #2	Patient #3	Patient #4	Patient #5	Patient #6
Procedural success	Yes	Yes	Yes	Yes	Yes	Yes
Procedural time (min)^*^	135.0	135.0	120.0	125.0	150.0	140.0
Device time (min)^†^	8.0	7.0	9.0	8.0	12.0	9.0
Fluoroscopy time (min)	12.0	12.0	15.0	14.0	19.0	15.0
Bleeding volume (ml)	40.0	30.0	50.0	50.0	100.0	80.0
**Intraoperative, postdevice TEE**						
Peak transtricuspid gradient (mm Hg)	22.0	12.0	17.0	9.0	23.0	22.0
Mean transtricuspid gradient (mm Hg)	8.0	5.0	6.0	4.0	8.0	7.0
Tricuspid valve area (cm^2^)	3.6	3.0	2.9	2.4	3.3	3.2
**Intraoperative complications**						
Conversion to median sternotomy	No	No	No	No	No	No
Right coronary injury	No	No	No	No	No	No
Perforation of right ventricle wall	No	No	No	No	No	No
New-onset third-degree atrioventricular block	No	No	No	No	No	No
**In-hospital outcomes**						
ICU length (days)	4.0	1.5	2.0	1.0	6.0	2.5
Postoperative hospitalization length (days)	14.0	11.0	10.0	8.0	17.0	10.0
Residual TR ≥ moderate^‡^	No	No	No	No	Yes	No
Postoperative 24 h chest drainage (ml)	600.0	150.0	400.0	300.0	750.0	500.0
Myocardial infarction	No	No	No	No	No	No
Renal failure requiring dialysis	No	No	No	No	No	No
Gastrointestinal hemorrhage	No	No	No	No	No	No
Device migration	No	No	No	No	No	No
Device thrombosis	No	No	No	No	No	No
Pulmonary embolism	No	No	No	No	No	No
Stroke/TIA	No	No	No	No	No	No
New-onset third-degree atrioventricular block	No	No	No	No	No	No

Values are presented as *n* (%) or median (25th, 75th percentile).

ICU, intensive care unit; TR, tricuspid regurgitation; TIA, transient ischemic attack.

^*^ Defined as the duration from initial skin incision to final wound closure.

^†^ Defined as the duration from guiding sheath insertion into the RA to retrieval of the delivery system.

^‡^ One was central regurgitation, and the others were perivalvular leakage.

### 2-year follow-up data

**Figure 7:**
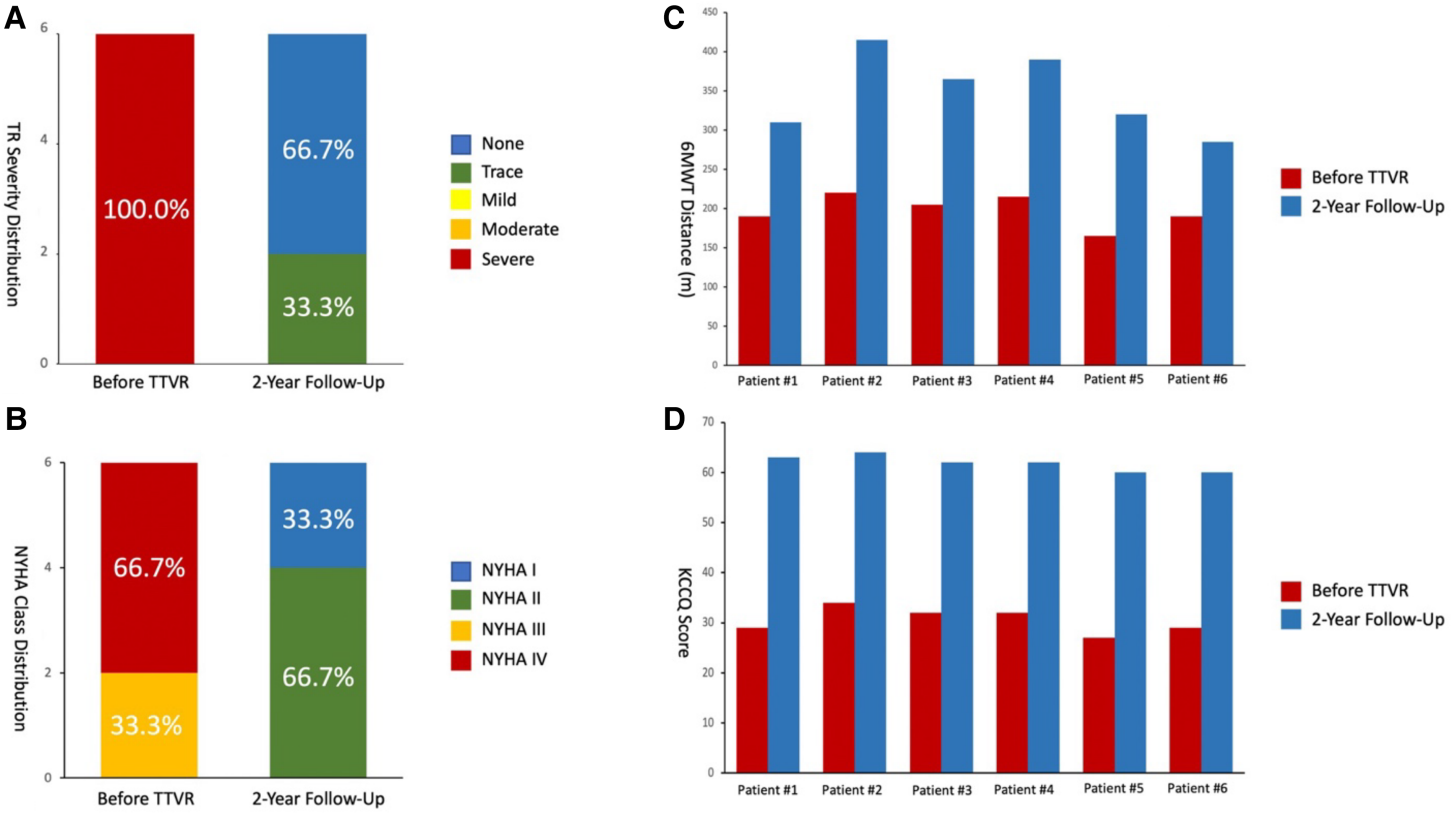
2-year follow-up results showed reduced TR severity and improved clinical, functional, and quality-of-life outcomes. (**A**) Assessment of TR severity. *P* value from Wilcoxon signed rank test. (**B**) Comparison of NYHA functional class between pre-procedures and 2-year follow-up. *P* value from Wilcoxon signed rank test. (**C**) Comparison of 6MWD distance. *P* value from paired Student's *t*-test. (**D**) Assessment of KCCQ score. *P* value from paired Student's *t*-test. TR, tricuspid regurgitation; 6MWD, 6-min walk distance; KCCQ, Kansas City cardiomyopathy questionnaire; NYHA, New York heart association.

Details of the 2-year follow-up data are shown in Table 5 and Figure 7. All patients showed significant improvement in symptoms at 6 months. TR severity measured by TTE decreased from 100.0% for severe regurgitation to 100.0% for no/trace regurgitation. The TV annulus diameter and RV length diameter were both decreased compared with the preoperative measurements, indicating RV remodeling. For the 1-year follow-up data, TR remained no/trace in all 6 patients (100.0%), and all patients showed improvement in NYHA class. For 6 patients with 2-year follow-up data, no/trace regurgitation occurred in all patients (100.0%, *P* = 0.014) (Figure 7A). In patients who had previously been implanted with a permanent pacemaker or implantable cardioverter defibrillator, the lead remained attached to the RV with no change in threshold after the valve was implanted. In addition, the reduction in TV ring diameter and the increased deviation of the TV annular plane in systole indicated improvement in RV structure and function. Two patients had NYHA functional class I, four patients had NYHA functional class II, and no device-related complications occurred (Figure 7B). The 6-min walking test (6MWT) showed significant improvement in motion performance (342.5 (305.0, 390.0) m vs. 200.0 (190.0, 215.0) m, *P* = 1.9  ×  10^−4^). Kansas City cardiomyopathy questionnaire (KCCQ) scores also improved significantly at 2-year follow-up (62.0 (60.0, 63.0) vs. 30.5 (29.0, 32.0), *P* = 5.5  ×  10^−8^) (Figures 7C, D).

**Table 5:** Baseline to 2-year echocardiographic measurements.

2-year follow-up outcomes	Patient #1		Patient #2		Patient #3		Patient #4		Patient #5		Patient #6	
Echocardiographic parameters	Pre	2-year	Pre	2-year	Pre	2-year	Pre	2-year	Pre	2-year	Pre	2-year
TR severity^*^	Severe	Trace	Severe	None	Severe	None	Severe	None	Severe	None	Severe	Trace
TAPSE (mm)	15.5	17.4	13.3	15.6	12.7	14.9	11.6	14.4	14.4	16.3	13.5	15.5
Fractional area change (%)	41.1	45.0	38.0	41.6	37.5	41.2	34.6	37.9	39.7	42.4	42.7	45.9
EROA PISA (mm^2^)	72.0	–	68.0	–	71.0	–	67.0	–	75.0	–	70.0	–
Mean transtricuspid gradient (mm Hg)	2.8	2.9	1.8	1.9	2.0	2.2	1.4	1.5	3.0	3.0	2.7	2.8
RV basal diameter (mm)	66.5	56.6	55.3	50.7	52.8	48.9	49.3	44.8	62.0	53.9	57.8	52.6
RV mid diameter (mm)	48.8	43.3	43.0	36.9	41.5	35.4	38.0	31.7	50.5	40.1	46.3	39.5
RV volume (ml)	93.5	68.5	82.3	62.6	80.8	59.3	73.0	52.1	87.0	66.9	84.5	60.3
RA volume (ml)	234.0	201.0	188.0	157.4	167.8	131.5	143.5	119.7	218.3	193.1	203.5	182.6
LV volume (ml)	117.0	104.3	93.3	83.8	84.3	77.0	73.8	70.5	105.3	98.7	96.0	90.7
LA volume (ml)	151.0	139.2	125.8	111.6	110.5	106.1	96.8	89.5	136.5	129.8	130.8	113.4
LVEF (%)	54.0	55.0	46.0	48.0	44.0	49.0	46.0	48.0	43.0	44.0	52.0	53.0

Values are presented as *N* (%) or median (25th, 75th percentile).

EROA, effective regurgitation orifice area; LA, left atrium; LV, left ventricle; PISA, proximal isovelocity surface area; RA, right atrium; RV, right ventricle; TAPSE, tricuspid annular plane systolic excursion; TR, tricuspid regurgitation; LVEF, left ventricular ejection fraction.

^*^ TR severity is classified into mild, moderate, severe, very severe and extremely severe (21). Postoperative residual TR was all none or trace perivalvular leakage except for one patient who had mild central regurgitation at postoperative 2 years.

## Discussion

In this single-center and observational study, LuX-Valve was successfully implanted in all 6 patients, and good clinical treatment results were achieved without complex TV anatomical structures and different etiologies. The unique anatomical structures and pathophysiological characteristics of TV make the design of TTVR devices difficult. From a physiological perspective, TV is a three-dimensional structure similar to a saddle, which has dynamic changes during the cardiac cycle to ensure complete valve closure. Primary TR is caused by congenital or acquired abnormalities of TV itself. However, secondary (or functional) TR, which is far more common than primary TR, is secondary to excess RV pressure and/or volume load. When TR occurs, TV loses its normal shape and dilates under the strain of the dilated RA and RV. Recent studies suggest that overloading of the RV caused by long-term TR may lead to irreversible myocardial injury of the RV (17).

In addition, with PPI being widely used in the treatment of chronic arrhythmia, complications caused by PPI have an increasing trend (1, 2, 18). Nachnani et al. (19) reported TR after PPI for the first time in 1969 but failed to identify the etiology. In 1972, Fishenfeld and Lamy (20) proposed the first etiological hypothesis of LITR. However, due to the small number of cases, there were significant differences in the results and therapeutic measures (18, 21). According to a number of studies, the common causes of TR caused by PPI mainly include lead impingement on the leaflets, adhesion between the lead and TV, lead wind and perforation. Among them, the incidence of lead impingement was 25%–47%, which occurred in 39% of patients with severe LITR, and all these factors directly resulted in incomplete closure (4, 6, 15). Some researchers have suggested that the period of PPI, the number of electrodes, the preprocedural size of RA and mild MR or TR before procedures are the relevant factors affecting long-term severe TR after PPI (15, 22). Additional studies have shown that electrical factors, such as delayed excitation of the RV, geometric changes in the RV, or mechanical dyssynchronization, may also cause TR (15, 22).

As a result, as the focus of LITR has increased, the amount of TV surgery has increased (17). Most studies have reported incomplete reduction of TR (23). A recent large registry of TAVR showed that preoperative TR severity was independently associated with 1-year postoperative mortality and heart failure rehospitalization (24). In general, TV may not provide stable support for traditional radial TTVR devices.

At present, MitraClip is the most widely used device for LITR, but residual TR could still be observed in postprocedural follow-up, and its long-term outcomes have not been clear. Regazzoli et al. (25) reported for the first time in 2017 that MitraClip (Abbott Vessels, Santa Clara, California) was used to repair a patient with LITR. Subsequently, Taramasso et al. (26) analyzed the clinical data of 470 patients with severe symptomatic TR included in the clinical registry of TriValve and found that 87% of patients in the PPI group received MitraClip, with a success rate of 78.6%, a mortality rate of 3.7% in the hospital, and residual TR ≤ 2 + in 73.7% of patients at the 30-day follow-up. In addition, Paradis et al. (27) in 2015 treated LITR by using Edwards Sapien XT (Edwards Life Sciences, Irvine, California), in which pacemaker leads were fixed between the two leaflets and performed well. Anderson et al. (28) analyzed the clinical data of 329 patients in the VIVID database, and 58 patients had PPI previously. As the first publicly reported TV, the Gate System (NaviGate Cardiac Structures, Inc., Lake Forest, California) has received limited reports for LITR and was not used in patients with TR caused by pacemaker leads (29). LuX-Valve is an *in situ* TTVR device with a nonradial support force, which has unique advantages compared with the traditional radial support force device. Its valve size selection is based on the effective orifice area rather than the expanded TV, which makes the diameter selection smaller. This design also ensures that the annulus diameter decreases as RV remodeling reverses. In addition, the smaller valve has no radial support on TV, so it is almost impossible to induce right ventricular outflow tract (RVOT) obstruction, right coronary artery injury or conduction block (7). LuX-Valve has a larger atrial plate than other devices, which effectively prevents paravalvular leakage after implantation. These advantages suggest that LuX-Valve is suitable for the treatment of TR caused by a variety of etiologies, including functional TR, TR caused by the pacemaker lead and by chronic AF.

Additionally, cardiovascular 3D printing has achieved great progress. Composite materials, such as different hardnesses of silica gel, resin, polyethylene and rubber, were used to print out the structures to achieve the real level of anatomical reduction. Patient-specific 3D printed models of tricuspid anatomy can facilitate preprocedural planning, educate patients and clinicians, and improve device design, leading to the overall improvement of patient outcomes and care. 3D printing represents an additional tool for more comprehensive preprocedural planning of tricuspid interventions and observation of tricuspid valve geometry. Vukicevic et al. (30) reported that tricuspid replicas allow benchtop observation of an individual patient's anatomy, device implantation in physiological TVs and interactions of devices with native tricuspid tissue, frequently leading to optimization or change in operational strategy. Wang et al. (31) suggested that the incorporation of CT and cardiac magnetic resonance imaging will likely provide increasing accuracy and optimization of transcatheter repair success. Overall, cardiovascular 3D printing may help surgeons choose the types of bioprostheses, significantly improve the success rate of TTVR and reduce the incidence of postoperative complications. Due to advancements in cardiovascular imaging and interventional technologies, tricuspid valve repairs and replacement interventions have become increasingly more attainable.

### Limitations

The study has some limitations. First, this study lacks a control group of traditional surgery with a small sample size, which requires a larger sample size and well-designed clinical trial to confirm its long-term safety and effectiveness. Second, the limitation of LuX-Valve is that the surgical approach is still through thoracic incision, and its delivery system needs to be further improved to be implanted through the peripheral vein path. Third, the evaluation of the manufacturing process of 3D printed models both cost massive funds and are time-consuming. At present, the above technologies are only carried out in large centers. In addition, the follow-up time was limited.

## Conclusions

In this study, patients with severe functional TR were treated by TTVR, which with the guidance of 3D printing, indicates a feasible, relatively safe and low-complication approach that improves RV remodeling and cardiac output with reliable clinical outcomes.

## Data Availability

The original contributions presented in the study are included in the article/Supplementary Material, further inquiries can be directed to the corresponding author/s.
